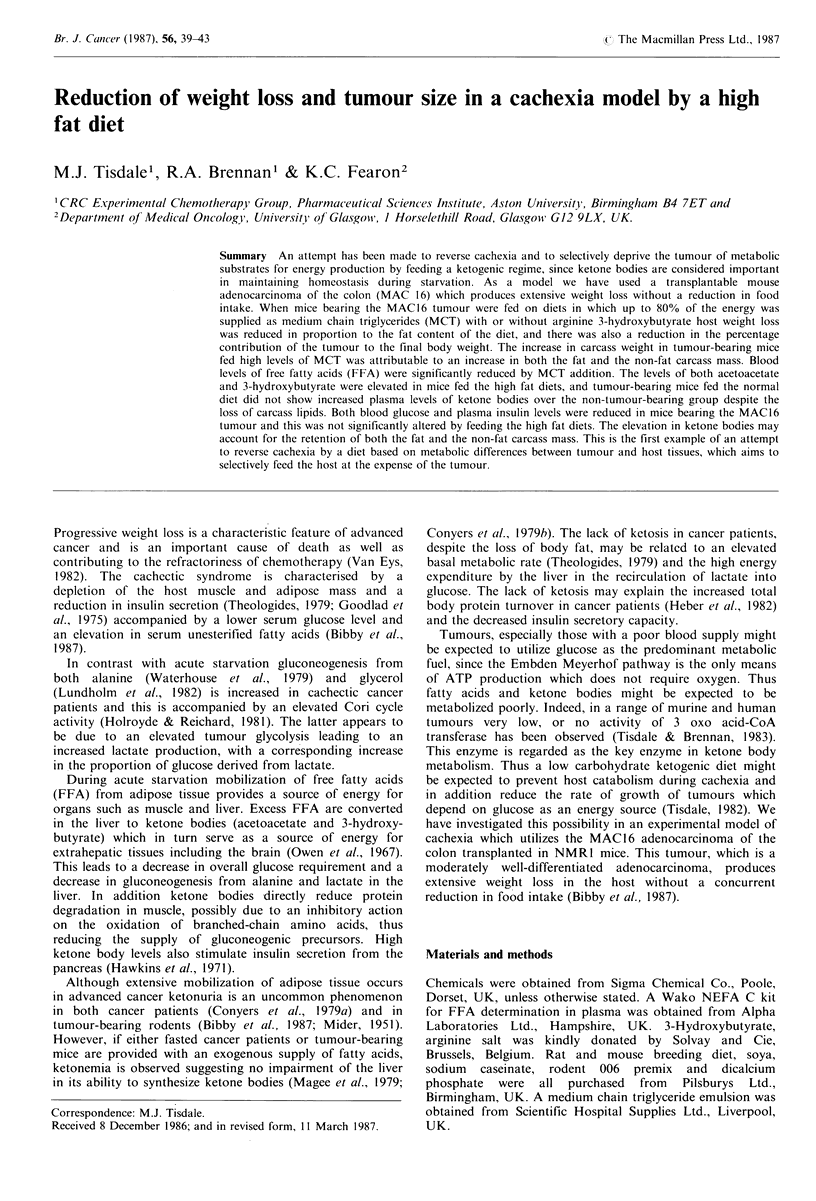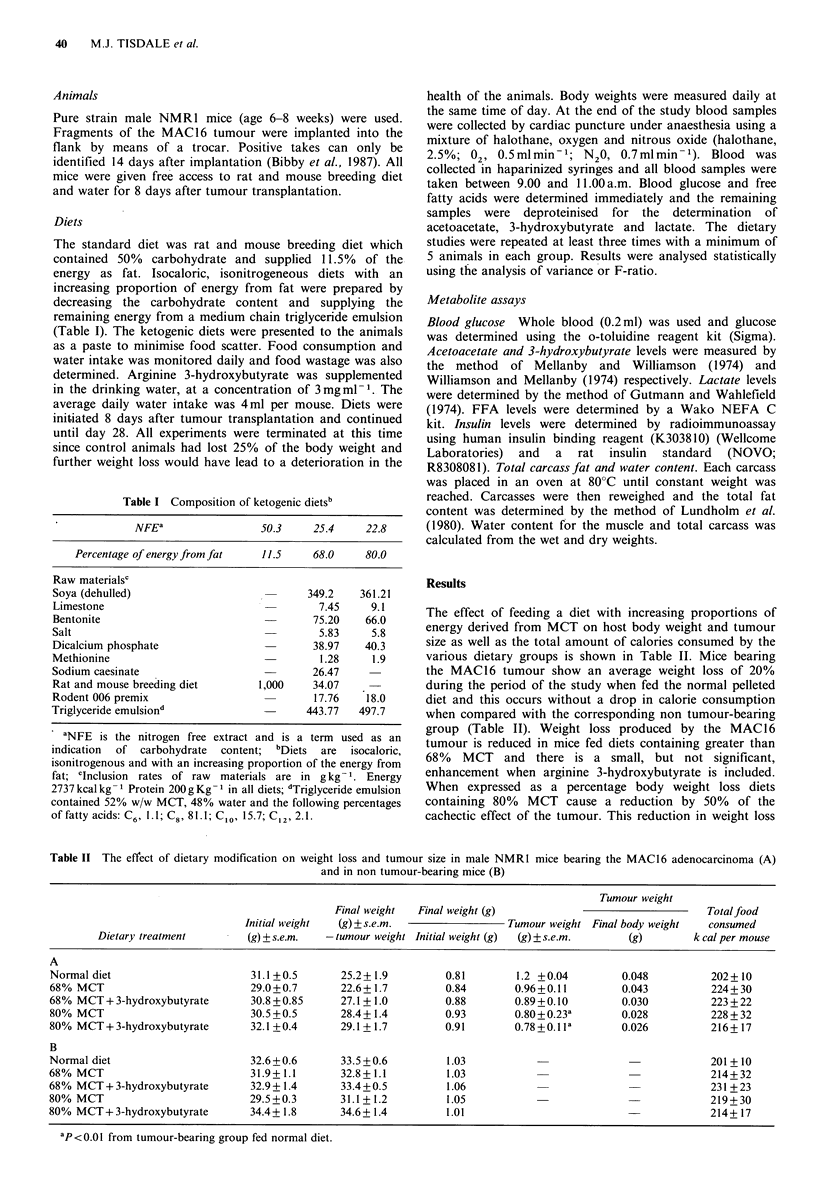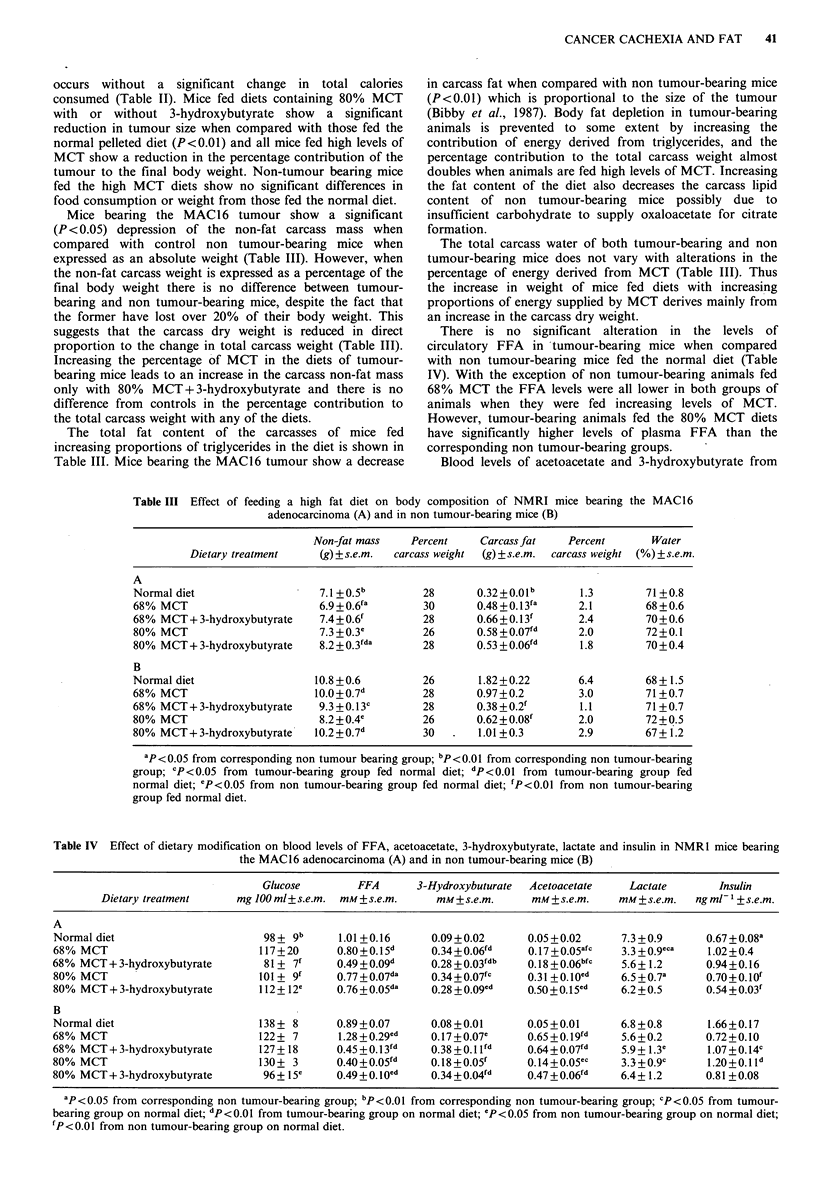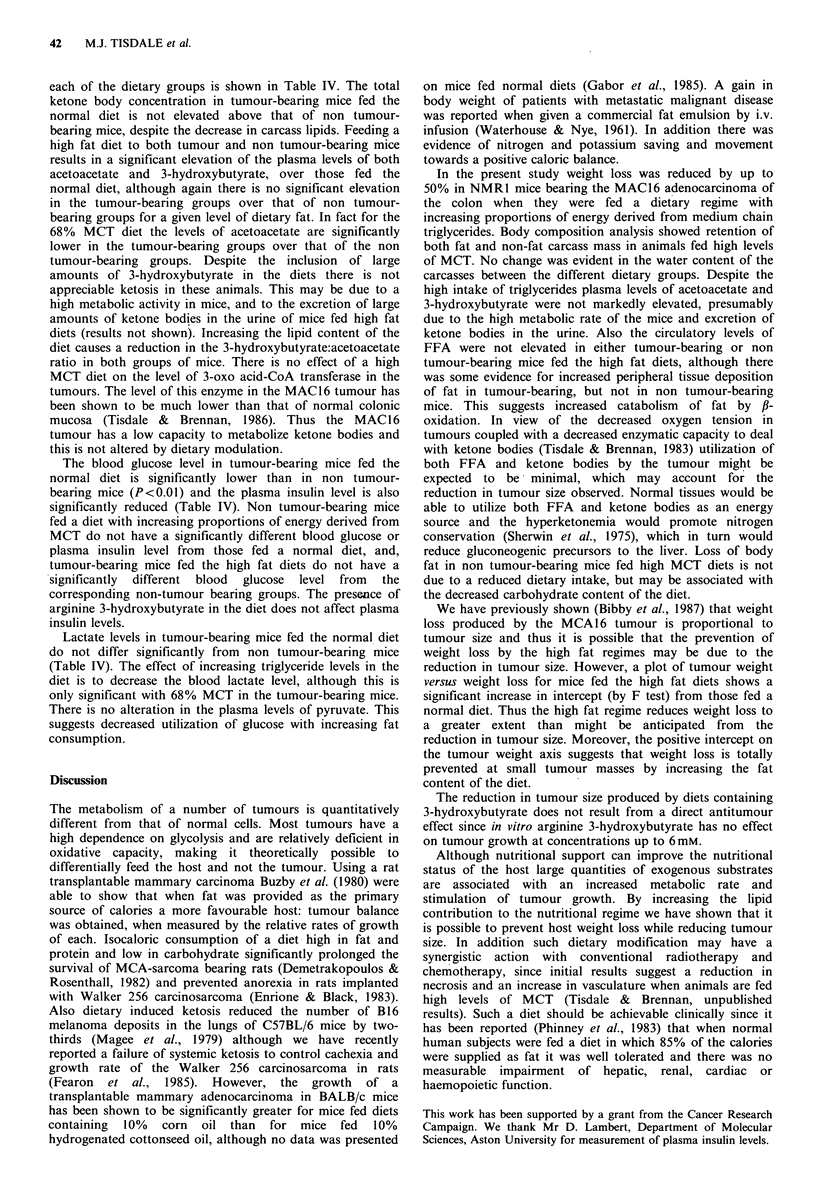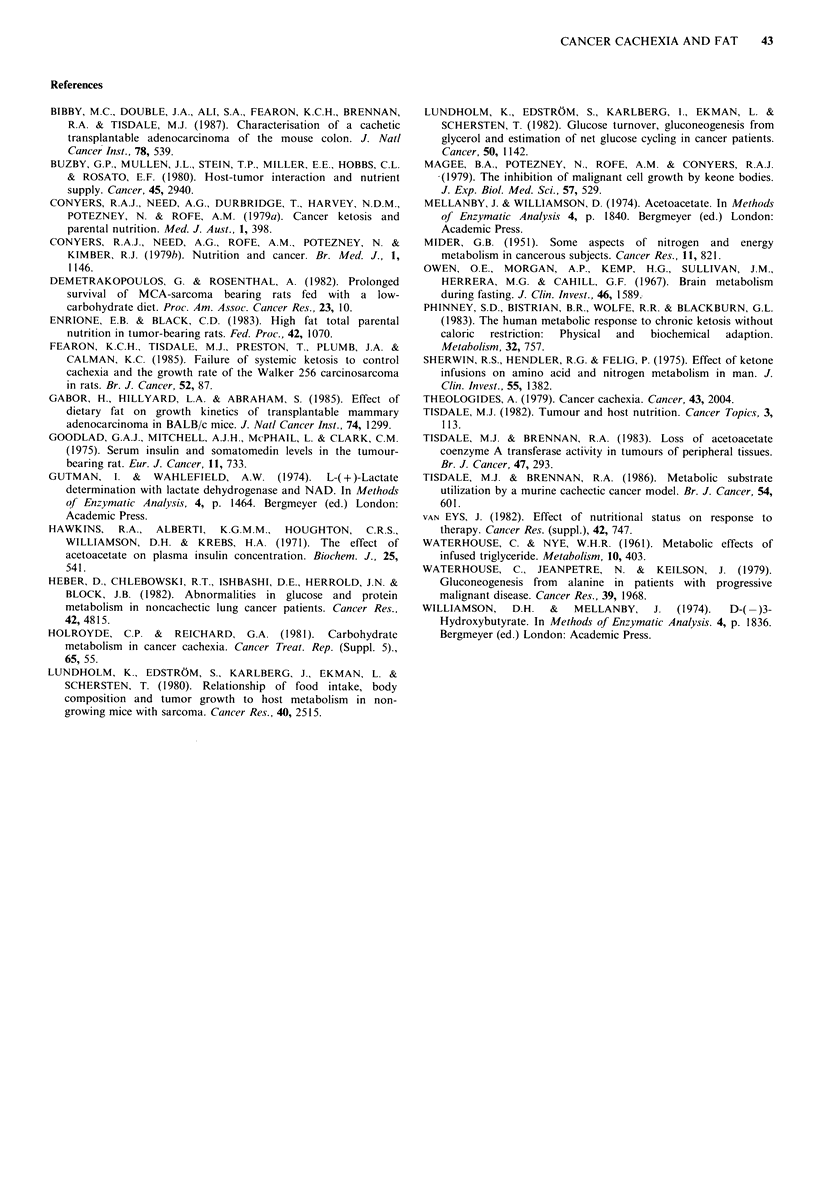# Reduction of weight loss and tumour size in a cachexia model by a high fat diet.

**DOI:** 10.1038/bjc.1987.149

**Published:** 1987-07

**Authors:** M. J. Tisdale, R. A. Brennan, K. C. Fearon

## Abstract

An attempt has been made to reverse cachexia and to selectively deprive the tumour of metabolic substrates for energy production by feeding a ketogenic regime, since ketone bodies are considered important in maintaining homeostasis during starvation. As a model we have used a transplantable mouse adenocarcinoma of the colon (MAC 16) which produces extensive weight loss without a reduction in food intake. When mice bearing the MAC16 tumour were fed on diets in which up to 80% of the energy was supplied as medium chain triglycerides (MCT) with or without arginine 3-hydroxybutyrate host weight loss was reduced in proportion to the fat content of the diet, and there was also a reduction in the percentage contribution of the tumour to the final body weight. The increase in carcass weight in tumour-bearing mice fed high levels of MCT was attributable to an increase in both the fat and the non-fat carcass mass. Blood levels of free fatty acids (FFA) were significantly reduced by MCT addition. The levels of both acetoacetate and 3-hydroxybutyrate were elevated in mice fed the high fat diets, and tumour-bearing mice fed the normal diet did not show increased plasma levels of ketone bodies over the non-tumour-bearing group despite the loss of carcass lipids. Both blood glucose and plasma insulin levels were reduced in mice bearing the MAC16 tumour and this was not significantly altered by feeding the high fat diets. The elevation in ketone bodies may account for the retention of both the fat and the non-fat carcass mass. This is the first example of an attempt to reverse cachexia by a diet based on metabolic differences between tumour and host tissues, which aims to selectively feed the host at the expense of the tumour.


*Br. J. Cancer* (1987), **56**, 39–43
© The Macmillan Press Ltd., 1987

### Reduction of weight loss and tumour size in a cachexia model by a high fat diet

M.J. Tisdale[1], R.A. Brennan[1] & K.C. Fearon[2]

[1]*CRC Experimental Chemotherapy Group, Pharmaceutical Sciences Institute, Aston University, Birmingham B4 7ET and* [2]*Department of Medical Oncology, University of Glasgow, 1 Horselethill Road, Glasgow G12 9LX, UK.*

**Summary** An attempt has been made to reverse cachexia and to selectively deprive the tumour of metabolic substrates for energy production by feeding a ketogenic regime, since ketone bodies are considered important in maintaining homeostasis during starvation. As a model we have used a transplantable mouse adenocarcinoma of the colon (MAC 16) which produces extensive weight loss without a reduction in food intake. When mice bearing the MAC16 tumour were fed on diets in which up to 80% of the energy was supplied as medium chain triglycerides (MCT) with or without arginine 3-hydroxybutyrate host weight loss was reduced in proportion to the fat content of the diet, and there was also a reduction in the percentage contribution of the tumour to the final body weight. The increase in carcass weight in tumour-bearing mice fed high levels of MCT was attributable to an increase in both the fat and the non-fat carcass mass. Blood levels of free fatty acids (FFA) were significantly reduced by MCT addition. The levels of both acetoacetate and 3-hydroxybutyrate were elevated in mice fed the high fat diets, and tumour-bearing mice fed the normal diet did not show increased plasma levels of ketone bodies over the non-tumour-bearing group despite the loss of carcass lipids. Both blood glucose and plasma insulin levels were reduced in mice bearing the MAC16 tumour and this was not significantly altered by feeding the high fat diets. The elevation in ketone bodies may account for the retention of both the fat and the non-fat carcass mass. This is the first example of an attempt to reverse cachexia by a diet based on metabolic differences between tumour and host tissues, which aims to selectively feed the host at the expense of the tumour.

Progressive weight loss is a characteristic feature of advanced cancer and is an important cause of death as well as contributing to the refractoriness of chemotherapy (Van Eys, 1982). The cachectic syndrome is characterised by a depletion of the host muscle and adipose mass and a reduction in insulin secretion (Theologides, 1979; Goodlad *et al.*, 1975) accompanied by a lower serum glucose level and an elevation in serum unesterified fatty acids (Bibby *et al.*, 1987).

In contrast with acute starvation gluconeogenesis from both alanine (Waterhouse *et al.*, 1979) and glycerol (Lundholm *et al.*, 1982) is increased in cachectic cancer patients and this is accompanied by an elevated Cori cycle activity (Holroyde & Reichard, 1981). The latter appears to be due to an elevated tumour glycolysis leading to an increased lactate production, with a corresponding increase in the proportion of glucose derived from lactate.

During acute starvation mobilization of free fatty acids (FFA) from adipose tissue provides a source of energy for organs such as muscle and liver. Excess FFA are converted in the liver to ketone bodies (acetoacetate and 3-hydroxybutyrate) which in turn serve as a source of energy for extrahepatic tissues including the brain (Owen *et al.*, 1967). This leads to a decrease in overall glucose requirement and a decrease in gluconeogenesis from alanine and lactate in the liver. In addition ketone bodies directly reduce protein degradation in muscle, possibly due to an inhibitory action on the oxidation of branched-chain amino acids, thus reducing the supply of gluconeogenic precursors. High ketone body levels also stimulate insulin secretion from the pancreas (Hawkins *et al.*, 1971).

Although extensive mobilization of adipose tissue occurs in advanced cancer ketonuria is an uncommon phenomenon in both cancer patients (Conyers *et al.*, 1979*a*) and in tumour-bearing rodents (Bibby *et al.*, 1987; Mider, 1951). However, if either fasted cancer patients or tumour-bearing mice are provided with an exogenous supply of fatty acids, ketonemia is observed suggesting no impairment of the liver in its ability to synthesize ketone bodies (Magee *et al.*, 1979; Conyers *et al.*, 1979*b*). The lack of ketosis in cancer patients, despite the loss of body fat, may be related to an elevated basal metabolic rate (Theologides, 1979) and the high energy expenditure by the liver in the recirculation of lactate into glucose. The lack of ketosis may explain the increased total body protein turnover in cancer patients (Heber *et al.*, 1982) and the decreased insulin secretory capacity.

Tumours, especially those with a poor blood supply might be expected to utilize glucose as the predominant metabolic fuel, since the Embden Meyerhof pathway is the only means of ATP production which does not require oxygen. Thus fatty acids and ketone bodies might be expected to be metabolized poorly. Indeed, in a range of murine and human tumours very low, or no activity of 3 oxo acid-CoA transferase has been observed (Tisdale & Brennan, 1983). This enzyme is regarded as the key enzyme in ketone body metabolism. Thus a low carbohydrate ketogenic diet might be expected to prevent host catabolism during cachexia and in addition reduce the rate of growth of tumours which depend on glucose as an energy source (Tisdale, 1982). We have investigated this possibility in an experimental model of cachexia which utilizes the MAC16 adenocarcinoma of the colon transplanted in NMR1 mice. This tumour, which is a moderately well-differentiated adenocarcinoma, produces extensive weight loss in the host without a concurrent reduction in food intake (Bibby *et al.*, 1987).

### Materials and methods

Chemicals were obtained from Sigma Chemical Co., Poole, Dorset, UK, unless otherwise stated. A Wako NEFA C kit for FFA determination in plasma was obtained from Alpha Laboratories Ltd., Hampshire, UK. 3-Hydroxybutyrate, arginine salt was kindly donated by Solvay and Cie, Brussels, Belgium. Rat and mouse breeding diet, soya, sodium caseinate, rodent 006 premix and dicalcium phosphate were all purchased from Pilsburys Ltd., Birmingham, UK. A medium chain triglyceride emulsion was obtained from Scientific Hospital Supplies Ltd., Liverpool, UK.

Correspondence: M.J. Tisdale.
Received 8 December 1986; and in revised form, 11 March 1987.

*Animals*

Pure strain male NMR1 mice (age 6–8 weeks) were used. Fragments of the MAC16 tumour were implanted into the flank by means of a trocar. Positive takes can only be identified 14 days after implantation (Bibby *et al.*, 1987). All mice were given free access to rat and mouse breeding diet and water for 8 days after tumour transplantation.

*Diets*

The standard diet was rat and mouse breeding diet which contained 50% carbohydrate and supplied 11.5% of the energy as fat. Isocaloric, isonitrogeneous diets with an increasing proportion of energy from fat were prepared by decreasing the carbohydrate content and supplying the remaining energy from a medium chain triglyceride emulsion (Table I). The ketogenic diets were presented to the animals as a paste to minimise food scatter. Food consumption and water intake was monitored daily and food wastage was also determined. Arginine 3-hydroxybutyrate was supplemented in the drinking water, at a concentration of $3\,mg\,ml^{-1}$. The average daily water intake was 4 ml per mouse. Diets were initiated 8 days after tumour transplantation and continued until day 28. All experiments were terminated at this time since control animals had lost 25% of the body weight and further weight loss would have lead to a deterioration in the health of the animals. Body weights were measured daily at the same time of day. At the end of the study blood samples were collected by cardiac puncture under anaesthesia using a mixture of halothane, oxygen and nitrous oxide (halothane, 2.5%; $0_2$, $0.5\,ml\,min^{-1}$; $N_20$, $0.7\,ml\,min^{-1}$). Blood was collected in haparinized syringes and all blood samples were taken between 9.00 and 11.00 a.m. Blood glucose and free fatty acids were determined immediately and the remaining samples were deproteinised for the determination of acetoacetate, 3-hydroxybutyrate and lactate. The dietary studies were repeated at least three times with a minimum of 5 animals in each group. Results were analysed statistically using the analysis of variance or F-ratio.

**Table I** Composition of ketogenic diets[b]

| *NFE*[a] | *50.3* | *25.4* | *22.8* |
|---|---|---|---|
| *Percentage of energy from fat* | *11.5* | *68.0* | *80.0* |
| Raw materials[c] | | | |
| Soya (dehulled) | — | 349.2 | 361.21 |
| Limestone | — | 7.45 | 9.1 |
| Bentonite | — | 75.20 | 66.0 |
| Salt | — | 5.83 | 5.8 |
| Dicalcium phosphate | — | 38.97 | 40.3 |
| Methionine | — | 1.28 | 1.9 |
| Sodium caesinate | — | 26.47 | — |
| Rat and mouse breeding diet | 1,000 | 34.07 | — |
| Rodent 006 premix | — | 17.76 | 18.0 |
| Triglyceride emulsion[d] | — | 443.77 | 497.7 |

[a]NFE is the nitrogen free extract and is a term used as an indication of carbohydrate content; [b]Diets are isocaloric, isonitrogenous and with an increasing proportion of the energy from fat; [c]Inclusion rates of raw materials are in $g\,kg^{-1}$. Energy $2737\,kcal\,kg^{-1}$ Protein $200\,g\,Kg^{-1}$ in all diets; [d]Triglyceride emulsion contained 52% w/w MCT, 48% water and the following percentages of fatty acids: $C_6$, 1.1; $C_8$, 81.1; $C_{10}$, 15.7; $C_{12}$, 2.1.

*Metabolite assays*

*Blood glucose* Whole blood (0.2 ml) was used and glucose was determined using the o-toluidine reagent kit (Sigma). *Acetoacetate and 3-hydroxybutyrate* levels were measured by the method of Mellanby and Williamson (1974) and Williamson and Mellanby (1974) respectively. *Lactate* levels were determined by the method of Gutmann and Wahlefield (1974). FFA levels were determined by a Wako NEFA C kit. *Insulin* levels were determined by radioimmunoassay using human insulin binding reagent (K303810) (Wellcome Laboratories) and a rat insulin standard (NOVO; R8308081). *Total carcass fat and water content.* Each carcass was placed in an oven at 80°C until constant weight was reached. Carcasses were then reweighed and the total fat content was determined by the method of Lundholm *et al.* (1980). Water content for the muscle and total carcass was calculated from the wet and dry weights.

### Results

The effect of feeding a diet with increasing proportions of energy derived from MCT on host body weight and tumour size as well as the total amount of calories consumed by the various dietary groups is shown in Table II. Mice bearing the MAC16 tumour show an average weight loss of 20% during the period of the study when fed the normal pelleted diet and this occurs without a drop in calorie consumption when compared with the corresponding non tumour-bearing group (Table II). Weight loss produced by the MAC16 tumour is reduced in mice fed diets containing greater than 68% MCT and there is a small, but not significant, enhancement when arginine 3-hydroxybutyrate is included. When expressed as a percentage body weight loss diets containing 80% MCT cause a reduction by 50% of the cachectic effect of the tumour. This reduction in weight loss

**Table II** The effect of dietary modification on weight loss and tumour size in male NMR1 mice bearing the MAC16 adenocarcinoma (A) and in non tumour-bearing mice (B)

| *Dietary treatment* | *Initial weight (g) ± s.e.m.* | *Final weight (g) ± s.e.m. − tumour weight* | *Final weight (g) / Initial weight (g)* | *Tumour weight (g) ± s.e.m.* | *Tumour weight / Final body weight (g)* | *Total food consumed k cal per mouse* |
|---|---|---|---|---|---|---|
| A | | | | | | |
| Normal diet | 31.1 ± 0.5 | 25.2 ± 1.9 | 0.81 | 1.2 ± 0.04 | 0.048 | 202 ± 10 |
| 68% MCT | 29.0 ± 0.7 | 22.6 ± 1.7 | 0.84 | 0.96 ± 0.11 | 0.043 | 224 ± 30 |
| 68% MCT + 3-hydroxybutyrate | 30.8 ± 0.85 | 27.1 ± 1.0 | 0.88 | 0.89 ± 0.10 | 0.030 | 223 ± 22 |
| 80% MCT | 30.5 ± 0.5 | 28.4 ± 1.4 | 0.93 | 0.80 ± 0.23[a] | 0.028 | 228 ± 32 |
| 80% MCT + 3-hydroxybutyrate | 32.1 ± 0.4 | 29.1 ± 1.7 | 0.91 | 0.78 ± 0.11[a] | 0.026 | 216 ± 17 |
| B | | | | | | |
| Normal diet | 32.6 ± 0.6 | 33.5 ± 0.6 | 1.03 | — | — | 201 ± 10 |
| 68% MCT | 31.9 ± 1.1 | 32.8 ± 1.1 | 1.03 | — | — | 214 ± 32 |
| 68% MCT + 3-hydroxybutyrate | 32.9 ± 1.4 | 33.4 ± 0.5 | 1.06 | — | — | 231 ± 23 |
| 80% MCT | 29.5 ± 0.3 | 31.1 ± 1.2 | 1.05 | — | — | 219 ± 30 |
| 80% MCT + 3-hydroxybutyrate | 34.4 ± 1.8 | 34.6 ± 1.4 | 1.01 | | — | 214 ± 17 |

[a]$P < 0.01$ from tumour-bearing group fed normal diet.

occurs without a significant change in total calories consumed (Table II). Mice fed diets containing 80% MCT with or without 3-hydroxybutyrate show a significant reduction in tumour size when compared with those fed the normal pelleted diet ($P<0.01$) and all mice fed high levels of MCT show a reduction in the percentage contribution of the tumour to the final body weight. Non-tumour bearing mice fed the high MCT diets show no significant differences in food consumption or weight from those fed the normal diet.

Mice bearing the MAC16 tumour show a significant ($P<0.05$) depression of the non-fat carcass mass when compared with control non tumour-bearing mice when expressed as an absolute weight (Table III). However, when the non-fat carcass weight is expressed as a percentage of the final body weight there is no difference between tumour-bearing and non tumour-bearing mice, despite the fact that the former have lost over 20% of their body weight. This suggests that the carcass dry weight is reduced in direct proportion to the change in total carcass weight (Table III). Increasing the percentage of MCT in the diets of tumour-bearing mice leads to an increase in the carcass non-fat mass only with 80% MCT+3-hydroxybutyrate and there is no difference from controls in the percentage contribution to the total carcass weight with any of the diets.

The total fat content of the carcasses of mice fed increasing proportions of triglycerides in the diet is shown in Table III. Mice bearing the MAC16 tumour show a decrease in carcass fat when compared with non tumour-bearing mice ($P<0.01$) which is proportional to the size of the tumour (Bibby *et al.*, 1987). Body fat depletion in tumour-bearing animals is prevented to some extent by increasing the contribution of energy derived from triglycerides, and the percentage contribution to the total carcass weight almost doubles when animals are fed high levels of MCT. Increasing the fat content of the diet also decreases the carcass lipid content of non tumour-bearing mice possibly due to insufficient carbohydrate to supply oxaloacetate for citrate formation.

The total carcass water of both tumour-bearing and non tumour-bearing mice does not vary with alterations in the percentage of energy derived from MCT (Table III). Thus the increase in weight of mice fed diets with increasing proportions of energy supplied by MCT derives mainly from an increase in the carcass dry weight.

There is no significant alteration in the levels of circulatory FFA in tumour-bearing mice when compared with non tumour-bearing mice fed the normal diet (Table IV). With the exception of non tumour-bearing animals fed 68% MCT the FFA levels were all lower in both groups of animals when they were fed increasing levels of MCT. However, tumour-bearing animals fed the 80% MCT diets have significantly higher levels of plasma FFA than the corresponding non tumour-bearing groups.

Blood levels of acetoacetate and 3-hydroxybutyrate from

**Table III** Effect of feeding a high fat diet on body composition of NMRI mice bearing the MAC16 adenocarcinoma (A) and in non tumour-bearing mice (B)

| Dietary treatment | *Non-fat mass (g)±s.e.m.* | *Percent carcass weight* | *Carcass fat (g)±s.e.m.* | *Percent carcass weight* | *Water (%)±s.e.m.* |
|---|---|---|---|---|---|
| A | | | | | |
| Normal diet | 7.1±0.5[b] | 28 | 0.32±0.01[b] | 1.3 | 71±0.8 |
| 68% MCT | 6.9±0.6[fa] | 30 | 0.48±0.13[fa] | 2.1 | 68±0.6 |
| 68% MCT+3-hydroxybutyrate | 7.4±0.6[f] | 28 | 0.66±0.13[f] | 2.4 | 70±0.6 |
| 80% MCT | 7.3±0.3[e] | 26 | 0.58±0.07[fd] | 2.0 | 72±0.1 |
| 80% MCT+3-hydroxybutyrate | 8.2±0.3[fda] | 28 | 0.53±0.06[fd] | 1.8 | 70±0.4 |
| B | | | | | |
| Normal diet | 10.8±0.6 | 26 | 1.82±0.22 | 6.4 | 68±1.5 |
| 68% MCT | 10.0±0.7[d] | 28 | 0.97±0.2 | 3.0 | 71±0.7 |
| 68% MCT+3-hydroxybutyrate | 9.3±0.13[c] | 28 | 0.38±0.2[f] | 1.1 | 71±0.7 |
| 80% MCT | 8.2±0.4[e] | 26 | 0.62±0.08[f] | 2.0 | 72±0.5 |
| 80% MCT+3-hydroxybutyrate | 10.2±0.7[d] | 30 | 1.01±0.3 | 2.9 | 67±1.2 |

[a]$P<0.05$ from corresponding non tumour bearing group; [b]$P<0.01$ from corresponding non tumour-bearing group; [c]$P<0.05$ from tumour-bearing group fed normal diet; [d]$P<0.01$ from tumour-bearing group fed normal diet; [e]$P<0.05$ from non tumour-bearing group fed normal diet; [f]$P<0.01$ from non tumour-bearing group fed normal diet.

**Table IV** Effect of dietary modification on blood levels of FFA, acetoacetate, 3-hydroxybutyrate, lactate and insulin in NMR1 mice bearing the MAC16 adenocarcinoma (A) and in non tumour-bearing mice (B)

| Dietary treatment | *Glucose mg 100 ml±s.e.m.* | *FFA mM±s.e.m.* | *3-Hydroxybuturate mM±s.e.m.* | *Acetoacetate mM±s.e.m.* | *Lactate mM±s.e.m.* | *Insulin ng ml$^{-1}$±s.e.m.* |
|---|---|---|---|---|---|---|
| A | | | | | | |
| Normal diet | 98±9[b] | 1.01±0.16 | 0.09±0.02 | 0.05±0.02 | 7.3±0.9 | 0.67±0.08[a] |
| 68% MCT | 117±20 | 0.80±0.15[d] | 0.34±0.06[fd] | 0.17±0.05[afc] | 3.3±0.9[eca] | 1.02±0.4 |
| 68% MCT+3-hydroxybutyrate | 81±7[f] | 0.49±0.09[d] | 0.28±0.03[fdb] | 0.18±0.06[bfc] | 5.6±1.2 | 0.94±0.16 |
| 80% MCT | 101±9[f] | 0.77±0.07[da] | 0.34±0.07[fc] | 0.31±0.10[ed] | 6.5±0.7[a] | 0.70±0.10[f] |
| 80% MCT+3-hydroxybutyrate | 112±12[e] | 0.76±0.05[da] | 0.28±0.09[ed] | 0.50±0.15[ed] | 6.2±0.5 | 0.54±0.03[f] |
| B | | | | | | |
| Normal diet | 138±8 | 0.89±0.07 | 0.08±0.01 | 0.05±0.01 | 6.8±0.8 | 1.66±0.17 |
| 68% MCT | 122±7 | 1.28±0.29[ed] | 0.17±0.07[e] | 0.65±0.19[fd] | 5.6±0.2 | 0.72±0.10 |
| 68% MCT+3-hydroxybutyrate | 127±18 | 0.45±0.13[fd] | 0.38±0.11[fd] | 0.64±0.07[fd] | 5.9±1.3[e] | 1.07±0.14[c] |
| 80% MCT | 130±3 | 0.40±0.05[fd] | 0.18±0.05[f] | 0.14±0.05[ec] | 3.3±0.9[c] | 1.20±0.11[d] |
| 80% MCT+3-hydroxybutyrate | 96±15[e] | 0.49±0.10[ed] | 0.34±0.04[fd] | 0.47±0.06[fd] | 6.4±1.2 | 0.81±0.08 |

[a]$P<0.05$ from corresponding non tumour-bearing group; [b]$P<0.01$ from corresponding non tumour-bearing group; [c]$P<0.05$ from tumour-bearing group on normal diet; [d]$P<0.01$ from tumour-bearing group on normal diet; [e]$P<0.05$ from non tumour-bearing group on normal diet; [f]$P<0.01$ from non tumour-bearing group on normal diet.

each of the dietary groups is shown in Table IV. The total ketone body concentration in tumour-bearing mice fed the normal diet is not elevated above that of non tumour-bearing mice, despite the decrease in carcass lipids. Feeding a high fat diet to both tumour and non tumour-bearing mice results in a significant elevation of the plasma levels of both acetoacetate and 3-hydroxybutyrate, over those fed the normal diet, although again there is no significant elevation in the tumour-bearing groups over that of non tumour-bearing groups for a given level of dietary fat. In fact for the 68% MCT diet the levels of acetoacetate are significantly lower in the tumour-bearing groups over that of the non tumour-bearing groups. Despite the inclusion of large amounts of 3-hydroxybutyrate in the diets there is not appreciable ketosis in these animals. This may be due to a high metabolic activity in mice, and to the excretion of large amounts of ketone bodies in the urine of mice fed high fat diets (results not shown). Increasing the lipid content of the diet causes a reduction in the 3-hydroxybutyrate:acetoacetate ratio in both groups of mice. There is no effect of a high MCT diet on the level of 3-oxo acid-CoA transferase in the tumours. The level of this enzyme in the MAC16 tumour has been shown to be much lower than that of normal colonic mucosa (Tisdale & Brennan, 1986). Thus the MAC16 tumour has a low capacity to metabolize ketone bodies and this is not altered by dietary modulation.

The blood glucose level in tumour-bearing mice fed the normal diet is significantly lower than in non tumour-bearing mice ($P<0.01$) and the plasma insulin level is also significantly reduced (Table IV). Non tumour-bearing mice fed a diet with increasing proportions of energy derived from MCT do not have a significantly different blood glucose or plasma insulin level from those fed a normal diet, and, tumour-bearing mice fed the high fat diets do not have a significantly different blood glucose level from the corresponding non-tumour bearing groups. The presence of arginine 3-hydroxybutyrate in the diet does not affect plasma insulin levels.

Lactate levels in tumour-bearing mice fed the normal diet do not differ significantly from non tumour-bearing mice (Table IV). The effect of increasing triglyceride levels in the diet is to decrease the blood lactate level, although this is only significant with 68% MCT in the tumour-bearing mice. There is no alteration in the plasma levels of pyruvate. This suggests decreased utilization of glucose with increasing fat consumption.

### Discussion

The metabolism of a number of tumours is quantitatively different from that of normal cells. Most tumours have a high dependence on glycolysis and are relatively deficient in oxidative capacity, making it theoretically possible to differentially feed the host and not the tumour. Using a rat transplantable mammary carcinoma Buzby *et al.* (1980) were able to show that when fat was provided as the primary source of calories a more favourable host: tumour balance was obtained, when measured by the relative rates of growth of each. Isocaloric consumption of a diet high in fat and protein and low in carbohydrate significantly prolonged the survival of MCA-sarcoma bearing rats (Demetrakopoulos & Rosenthall, 1982) and prevented anorexia in rats implanted with Walker 256 carcinosarcoma (Enrione & Black, 1983). Also dietary induced ketosis reduced the number of B16 melanoma deposits in the lungs of C57BL/6 mice by two-thirds (Magee *et al.*, 1979) although we have recently reported a failure of systemic ketosis to control cachexia and growth rate of the Walker 256 carcinosarcoma in rats (Fearon *et al.*, 1985). However, the growth of a transplantable mammary adenocarcinoma in BALB/c mice has been shown to be significantly greater for mice fed diets containing 10% corn oil than for mice fed 10% hydrogenated cottonseed oil, although no data was presented on mice fed normal diets (Gabor *et al.*, 1985). A gain in body weight of patients with metastatic malignant disease was reported when given a commercial fat emulsion by i.v. infusion (Waterhouse & Nye, 1961). In addition there was evidence of nitrogen and potassium saving and movement towards a positive caloric balance.

In the present study weight loss was reduced by up to 50% in NMR1 mice bearing the MAC16 adenocarcinoma of the colon when they were fed a dietary regime with increasing proportions of energy derived from medium chain triglycerides. Body composition analysis showed retention of both fat and non-fat carcass mass in animals fed high levels of MCT. No change was evident in the water content of the carcasses between the different dietary groups. Despite the high intake of triglycerides plasma levels of acetoacetate and 3-hydroxybutyrate were not markedly elevated, presumably due to the high metabolic rate of the mice and excretion of ketone bodies in the urine. Also the circulatory levels of FFA were not elevated in either tumour-bearing or non tumour-bearing mice fed the high fat diets, although there was some evidence for increased peripheral tissue deposition of fat in tumour-bearing, but not in non tumour-bearing mice. This suggests increased catabolism of fat by $\beta$-oxidation. In view of the decreased oxygen tension in tumours coupled with a decreased enzymatic capacity to deal with ketone bodies (Tisdale & Brennan, 1983) utilization of both FFA and ketone bodies by the tumour might be expected to be minimal, which may account for the reduction in tumour size observed. Normal tissues would be able to utilize both FFA and ketone bodies as an energy source and the hyperketonemia would promote nitrogen conservation (Sherwin *et al.*, 1975), which in turn would reduce gluconeogenic precursors to the liver. Loss of body fat in non tumour-bearing mice fed high MCT diets is not due to a reduced dietary intake, but may be associated with the decreased carbohydrate content of the diet.

We have previously shown (Bibby *et al.*, 1987) that weight loss produced by the MCA16 tumour is proportional to tumour size and thus it is possible that the prevention of weight loss by the high fat regimes may be due to the reduction in tumour size. However, a plot of tumour weight *versus* weight loss for mice fed the high fat diets shows a significant increase in intercept (by F test) from those fed a normal diet. Thus the high fat regime reduces weight loss to a greater extent than might be anticipated from the reduction in tumour size. Moreover, the positive intercept on the tumour weight axis suggests that weight loss is totally prevented at small tumour masses by increasing the fat content of the diet.

The reduction in tumour size produced by diets containing 3-hydroxybutyrate does not result from a direct antitumour effect since *in vitro* arginine 3-hydroxybutyrate has no effect on tumour growth at concentrations up to 6 mM.

Although nutritional support can improve the nutritional status of the host large quantities of exogenous substrates are associated with an increased metabolic rate and stimulation of tumour growth. By increasing the lipid contribution to the nutritional regime we have shown that it is possible to prevent host weight loss while reducing tumour size. In addition such dietary modification may have a synergistic action with conventional radiotherapy and chemotherapy, since initial results suggest a reduction in necrosis and an increase in vasculature when animals are fed high levels of MCT (Tisdale & Brennan, unpublished results). Such a diet should be achievable clinically since it has been reported (Phinney *et al.*, 1983) that when normal human subjects were fed a diet in which 85% of the calories were supplied as fat it was well tolerated and there was no measurable impairment of hepatic, renal, cardiac or haemopoietic function.

This work has been supported by a grant from the Cancer Research Campaign. We thank Mr D. Lambert, Department of Molecular Sciences, Aston University for measurement of plasma insulin levels.

**References**

BIBBY, M.C., DOUBLE, J.A., ALI, S.A., FEARON, K.C.H., BRENNAN, R.A. & TISDALE, M.J. (1987). Characterisation of a cachetic transplantable adenocarcinoma of the mouse colon. *J. Natl Cancer Inst.*, **78,** 539.

BUZBY, G.P., MULLEN, J.L., STEIN, T.P., MILLER, E.E., HOBBS, C.L. & ROSATO, E.F. (1980). Host-tumor interaction and nutrient supply. *Cancer*, **45,** 2940.

CONYERS, R.A.J., NEED, A.G., DURBRIDGE, T., HARVEY, N.D.M., POTEZNEY, N. & ROFE, A.M. (1979*a*). Cancer ketosis and parental nutrition. *Med. J. Aust.*, **1,** 398.

CONYERS, R.A.J., NEED, A.G., ROFE, A.M., POTEZNEY, N. & KIMBER, R.J. (1979*b*). Nutrition and cancer. *Br. Med. J.*, **1,** 1146.

DEMETRAKOPOULOS, G. & ROSENTHAL, A. (1982). Prolonged survival of MCA-sarcoma bearing rats fed with a low-carbohydrate diet. *Proc. Am. Assoc. Cancer Res.*, **23,** 10.

ENRIONE, E.B. & BLACK, C.D. (1983). High fat total parental nutrition in tumor-bearing rats. *Fed. Proc.*, **42,** 1070.

FEARON, K.C.H., TISDALE, M.J., PRESTON, T., PLUMB, J.A. & CALMAN, K.C. (1985). Failure of systemic ketosis to control cachexia and the growth rate of the Walker 256 carcinosarcoma in rats. *Br. J. Cancer*, **52,** 87.

GABOR, H., HILLYARD, L.A. & ABRAHAM, S. (1985). Effect of dietary fat on growth kinetics of transplantable mammary adenocarcinoma in BALB/c mice. *J. Natl Cancer Inst.*, **74,** 1299.

GOODLAD, G.A.J., MITCHELL, A.J.H., McPHAIL, L. & CLARK, C.M. (1975). Serum insulin and somatomedin levels in the tumour-bearing rat. *Eur. J. Cancer*, **11,** 733.

GUTMAN, I. & WAHLEFIELD, A.W. (1974). L-(+)-Lactate determination with lactate dehydrogenase and NAD. In *Methods of Enzymatic Analysis*, **4,** p. 1464. Bergmeyer (ed.) London: Academic Press.

HAWKINS, R.A., ALBERTI, K.G.M.M., HOUGHTON, C.R.S., WILLIAMSON, D.H. & KREBS, H.A. (1971). The effect of acetoacetate on plasma insulin concentration. *Biochem. J.*, **25,** 541.

HEBER, D., CHLEBOWSKI, R.T., ISHBASHI, D.E., HERROLD, J.N. & BLOCK, J.B. (1982). Abnormalities in glucose and protein metabolism in noncachectic lung cancer patients. *Cancer Res.*, **42,** 4815.

HOLROYDE, C.P. & REICHARD, G.A. (1981). Carbohydrate metabolism in cancer cachexia. *Cancer Treat. Rep.* (Suppl. 5)., **65,** 55.

LUNDHOLM, K., EDSTRÖM, S., KARLBERG, J., EKMAN, L. & SCHERSTEN, T. (1980). Relationship of food intake, body composition and tumor growth to host metabolism in non-growing mice with sarcoma. *Cancer Res.*, **40,** 2515.

LUNDHOLM, K., EDSTRÖM, S., KARLBERG, I., EKMAN, L. & SCHERSTEN, T. (1982). Glucose turnover, gluconeogenesis from glycerol and estimation of net glucose cycling in cancer patients. *Cancer*, **50,** 1142.

MAGEE, B.A., POTEZNEY, N., ROFE, A.M. & CONYERS, R.A.J. (1979). The inhibition of malignant cell growth by keone bodies. *J. Exp. Biol. Med. Sci.*, **57,** 529.

MELLANBY, J. & WILLIAMSON, D. (1974). Acetoacetate. In *Methods of Enzymatic Analysis* **4,** p. 1840. Bergmeyer (ed.) London: Academic Press.

MIDER, G.B. (1951). Some aspects of nitrogen and energy metabolism in cancerous subjects. *Cancer Res.*, **11,** 821.

OWEN, O.E., MORGAN, A.P., KEMP, H.G., SULLIVAN, J.M., HERRERA, M.G. & CAHILL, G.F. (1967). Brain metabolism during fasting. *J. Clin. Invest.*, **46,** 1589.

PHINNEY, S.D., BISTRIAN, B.R., WOLFE, R.R. & BLACKBURN, G.L. (1983). The human metabolic response to chronic ketosis without caloric restriction: Physical and biochemical adaption. *Metabolism*, **32,** 757.

SHERWIN, R.S., HENDLER, R.G. & FELIG, P. (1975). Effect of ketone infusions on amino acid and nitrogen metabolism in man. *J. Clin. Invest.*, **55,** 1382.

THEOLOGIDES, A. (1979). Cancer cachexia. *Cancer*, **43,** 2004.

TISDALE, M.J. (1982). Tumour and host nutrition. *Cancer Topics*, **3,** 113.

TISDALE, M.J. & BRENNAN, R.A. (1983). Loss of acetoacetate coenzyme A transferase activity in tumours of peripheral tissues. *Br. J. Cancer*, **47,** 293.

TISDALE, M.J. & BRENNAN, R.A. (1986). Metabolic substrate utilization by a murine cachectic cancer model. *Br. J. Cancer*, **54,** 601.

VAN EYS, J. (1982). Effect of nutritional status on response to therapy. *Cancer Res.* (suppl.), **42,** 747.

WATERHOUSE, C. & NYE, W.H.R. (1961). Metabolic effects of infused triglyceride. *Metabolism*, **10,** 403.

WATERHOUSE, C., JEANPETRE, N. & KEILSON, J. (1979). Gluconeogenesis from alanine in patients with progressive malignant disease. *Cancer Res.*, **39,** 1968.

WILLIAMSON, D.H. & MELLANBY, J. (1974). D-(−)3-Hydroxybutyrate. In *Methods of Enzymatic Analysis*. **4,** p. 1836. Bergmeyer (ed.) London: Academic Press.